# Strengthen the sacral ligament and paravagina by equilibrium control severe pelvic organ prolapse

**DOI:** 10.3389/fsurg.2022.1054008

**Published:** 2023-01-10

**Authors:** Xin Zhao, Jumin Niu, Yansong Liu

**Affiliations:** Shenyang Women's and Children's Hospital, Shengyang, China

**Keywords:** pelvic organ prolapse, laparoscopic sacrocolpopexy, paravaginal repair, Cooper suspension, paravaginal defects

## Abstract

**Objective:**

To evaluate and analyze the clinical effect of the combination of laparoscopic sacrocolpopexy (LSC), sacral ligament fusion and vaginal suspension in the treatment of severe pelvic organ prolapse

**Methods:**

A total of 76 cases of patients with pelvic organ prolapse in our hospital between January 2010 to December 2020 were enrolled for research. They had been evaluated pre- and post-operative through pelvic organ prolapse quantification (POP-Q) system, Pelvic Floor Dysfunction Questionnaire Short Form (PFDI-20), Pelvic Floor Function Impact Questionnaire Short form (PFIQ-7), and the Sexual Function Questionnaire Score (PIQS-31).

**Results:**

All 76 patients went through the procedure successfully without any complications. None of the 76 cases had relapsed. Post-operational results of PFDI-20 and PFIQ-7 were evidently lower than pre-operational results, post-operational results of PIQS-31 were higher than before operation.

**Conclusions:**

For patients with severe pelvic organ prolapse,a balanced control of the pelvic floor centred on the preservation of the stereoscopic ring around the cervix through revascularization is significantly effective, and has no recurrence after surgery, high patient satisfaction, fewer postoperative complications. It is safe and reliable and worthy of clinical application and promotion.

## 1. Introduction

With the continuous improvement of people's living standards, pelvic floor dysfunction has been recognised as a common disease among middle-aged and older women. Pelvic floor dysfunction was classified by the WHO as one of the five most harmful diseases to mankind in 1990s. About 50% of women were found to have weakened pelvic floor supporting structures after their delivery, resulting in some degree of pelvic organ prolapse (POP). Medical support was necessary for 10%–20% of them (1, 2) and 20% needed pelvic operations (3). The incidence of stress urinary incontinence (SUI) for women over 65 is 35%, and that of POP for women over 60 is 25% (4). In the United States, there are 40,000 cases of PFD operations annually, which is half the amount of all gynaecological operations (1).

Laparoscopic sacral colpopexy (LSC) is recognised as the “gold standard” operation for level I defects, with a long-term success rate of 74%–98% (5–7). This procedure connects the uterus or the top of the vagina to the sacral longitudinal ligament with transplants. It is widely applied to patients, including young and sexually active individuals (8). However, LSC merely corrected a level I defect and can only function for non-severe vaginal side defects (9). Richardson et al. believed that 85%–90% of anterior vaginal prolapse cases are caused by defects of the tissues adjacent to the vaginal anterior walls (10). In 1912, White suggested that the true reason for bladder inflation is that the separation between the fascia and pelvic wall forms defects in the tissues adjacent to the vaginal anterior walls.

In this article, we demonstrate the combination of LSC, sacral ligament fusion and vaginal suspension in order to enhance levels I and II while strengthening level III with the original anterior–posterior colporrhaphy. This procedure follows the idea that structure determines function, and it controls pelvic organ prolapse equilibrium. About 76 patients were effectively treated by this procedure.

## 2. Information and methods

### 2.1. General information

From January 2010 to December 2020, LSC with sacral ligament fusion and vaginal suspension was performed on 76 patients with severe POP. Inclusion criteria are as follows: Stage III and IV [POP Quantification System (POP-Q)] scores; ability to tolerate laparoscopic surgery; and being fully informed of the risks of using mesh before surgery. Exclusion criteria are as follows: internal and surgical complications; inability to tolerate laparoscopic surgery; rejection of intrauterine mesh; uterine malignancy; bilateral attachments; malignant lesions; coagulation malfunction; and infectious diseases. All patients signed informed consent, which was in accordance with the ethics committee of the hospital.

### 2.2. Choices of procedure

A. First, operate by anterior colporrhaphy.
B. Second, perform laparoscopic surgery. According to the specific situation of the patient, there were three options: total hysterectomy, cervical reservation, or uterine preservation. Hysterectomisation included laparoscopic intrafascial hysterectomy (part of extrafascial hysterectomy) to maintain the integrity of the cardinal and uterosacral ligaments, followed by LSC, sacral ligament fusion, paravaginal suspension, anterior and posterior vaginal wall repair. Cervical reservation included laparoscopic subtotal hysterectomy, LSC, sacral ligament fusion, paravaginal suspension, and anterior and posterior vaginal wall repair. Uterine preservation included LSC, sacral ligament fusion, paravaginal suspension, and anterior and posterior vaginal wall repair.
C. Posterior vaginal prolapse was treated with conventional posterior colporrhaphy.
D. Patients with stress urinary incontinence underwent tension-free trans-obturator suburethral tape (TVT-O) at the same time.

### 2.3. Evaluation index

The duration of the operation, bleeding volume, venting time after the operation, time of catheter removal, residue urine volume, and periprocedural complications were recorded. Follow-up examinations were carried out at 3, 6, and 12 months and annually afterwards after the operation. During the follow-up examination, the improved conditions of pre- and post-operation symptoms, including abdominal pain, pelvic tenesmus, urination, sexual function, and the presence of vaginal lesions, were inquired. Meanwhile, the complete questionnaire about living quality serves as the evaluation index of subjective satisfaction (11), including PFDI-20, PFIQ-7, and PIQS-31. POP-Q score and pelvic ultrasound result on the size of the gap in the genital tract and bladder neck displacement were used as the evaluation index for objective satisfaction.

### 2.4. Operative principle

#### 2.4.1. Laparoscopic intrafascial hysterectomy

Bipolar electrocoagulation was used to cut the round ligament, open the bladder-reflexed peritoneum, push the bladder down to below the level of the external opening of the cervical, separate the surrounding connective tissue, and expose the bilateral uterine arteries. Bipolar electrocoagulation was also used to cut the bilateral uterine arteries, push down the cervical fascia to expose the vaginal vault, and make a circular incision along the vaginal vault.

#### 2.4.2. Laparoscopic sacral colpopexy and sacral ligament fusion

**Figure 1:**
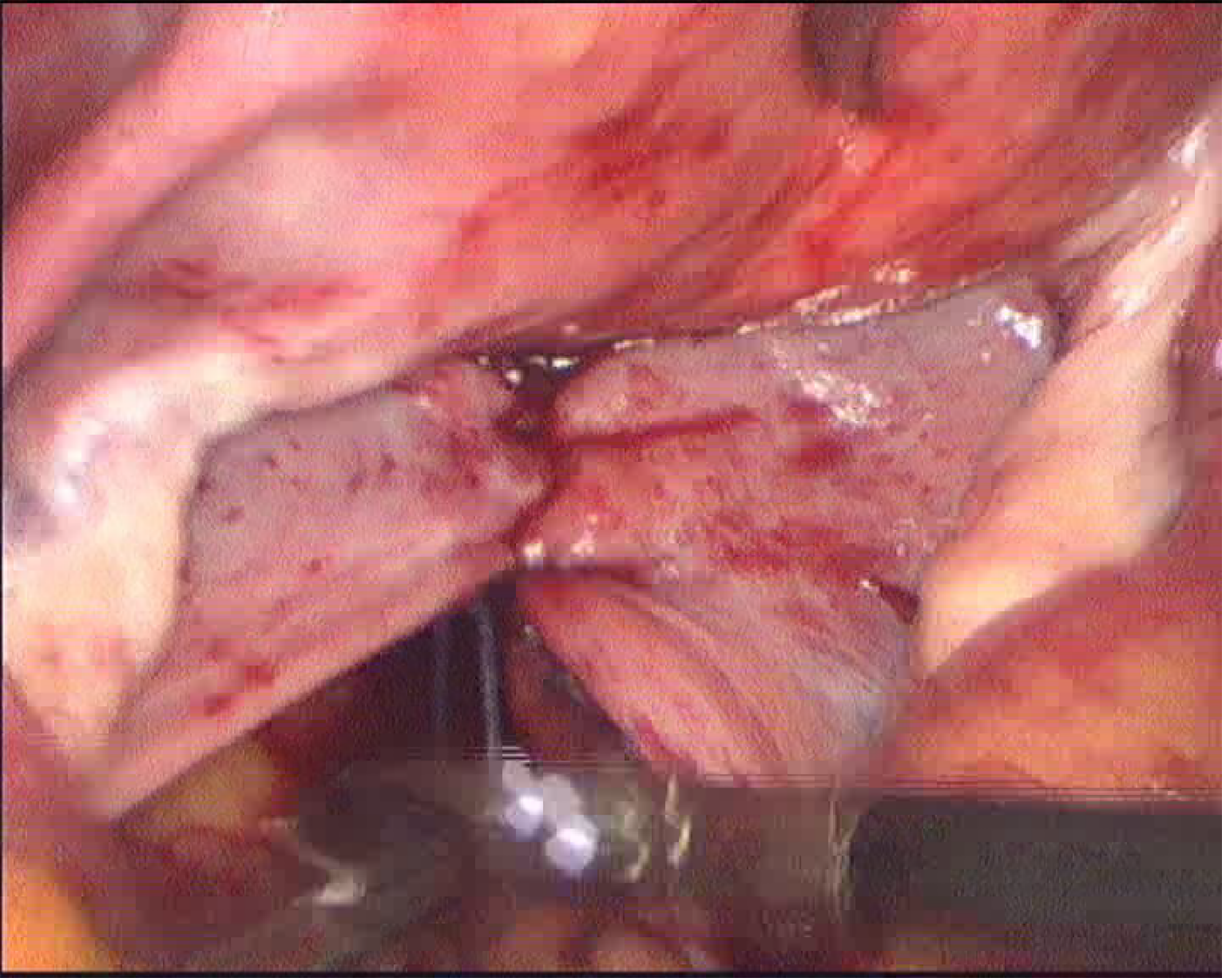
Sacral ligament fusion.

The technique includes general anaesthesia, lithotomy position, a catheter, umbilical puncture, establishing pneumoperitoneum at 11 mmHg, and embedding a 10-mm-diameter trocar, a peritoneoscope, and two 5-mm-diameter trocars at the left and right quadrants, respectively. Based on the choice of procedure made beforehand, a subtotal hysterectomy or an intrafascial hysterectomy was performed and then the following procedure was carried out. At the section on the bilateral iliosacral ligament where the cervix starts, the posterior peritoneum was opened horizontally. We opened the posterior peritoneum towards the promontory along the outer edge of the right side of the rectum, exposed the ureter and iliac vessels, separated the promontory connective tissue, exposed the anterior longitudinal ligament, and used non-absorbable lines to suture the short arm (2 cm long and 1.5 cm wide) of L-shaped meshes (Gynecare Gynemesh) at the rear of cervix where the iliosacral ligament starts. The long arm (16 cm long and 1.5 cm wide) was embedded at the outer edge of the rectum inside the interior gap of the opened iliosacral ligament. The long arm was sutured with the longitudinal ligament in the front of the promontory. Tension-free meshes were implanted into the iliosacral ligament and excessive meshes were cut off. The posterior peritoneum was sutured with absorbable lines, allowing peritonization of the pelvic cavity. Non-absorbable lines were used to put three to four stitches and fix bilateral iliosacral ligaments along the interior of the iliosacral ligaments to strengthen their fusion (Figure 1).

#### 2.4.3. Cooper suspension

**Figure 2:**
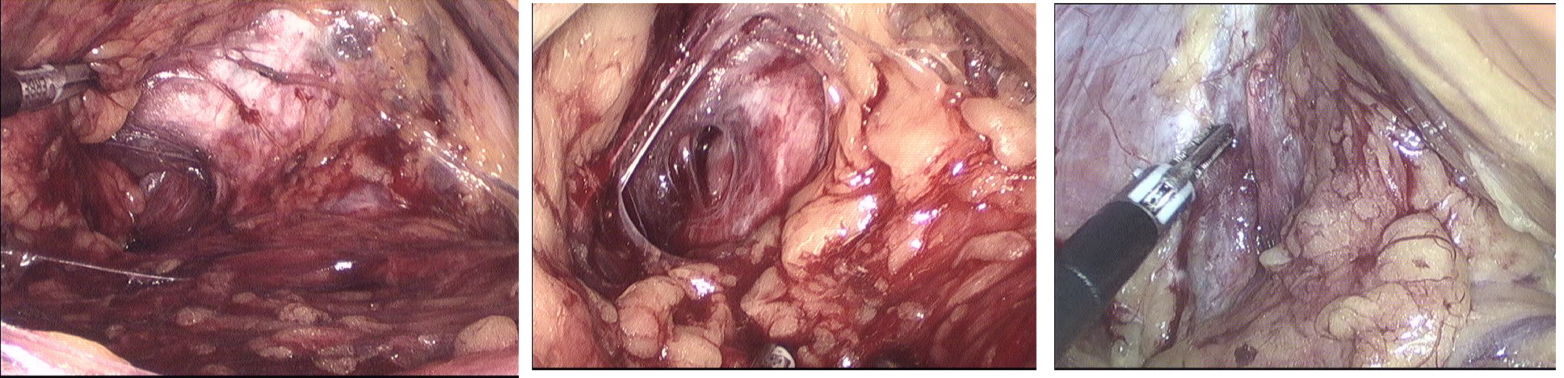
ATFP defect, separated from levator ani muscle, and anal levator fissure.

**Figure 3:**
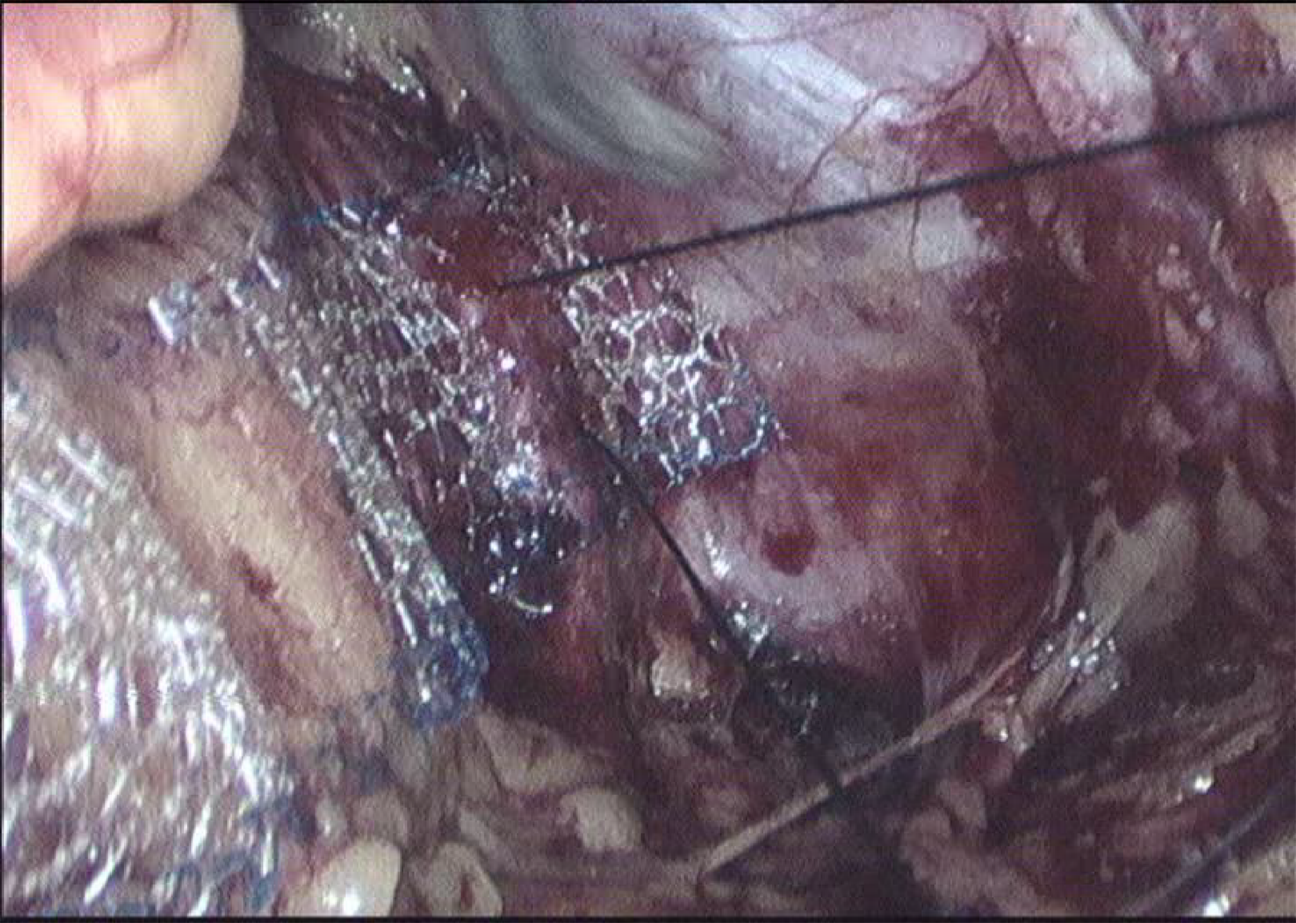
Mesh covering the gap.

**Figure 4:**
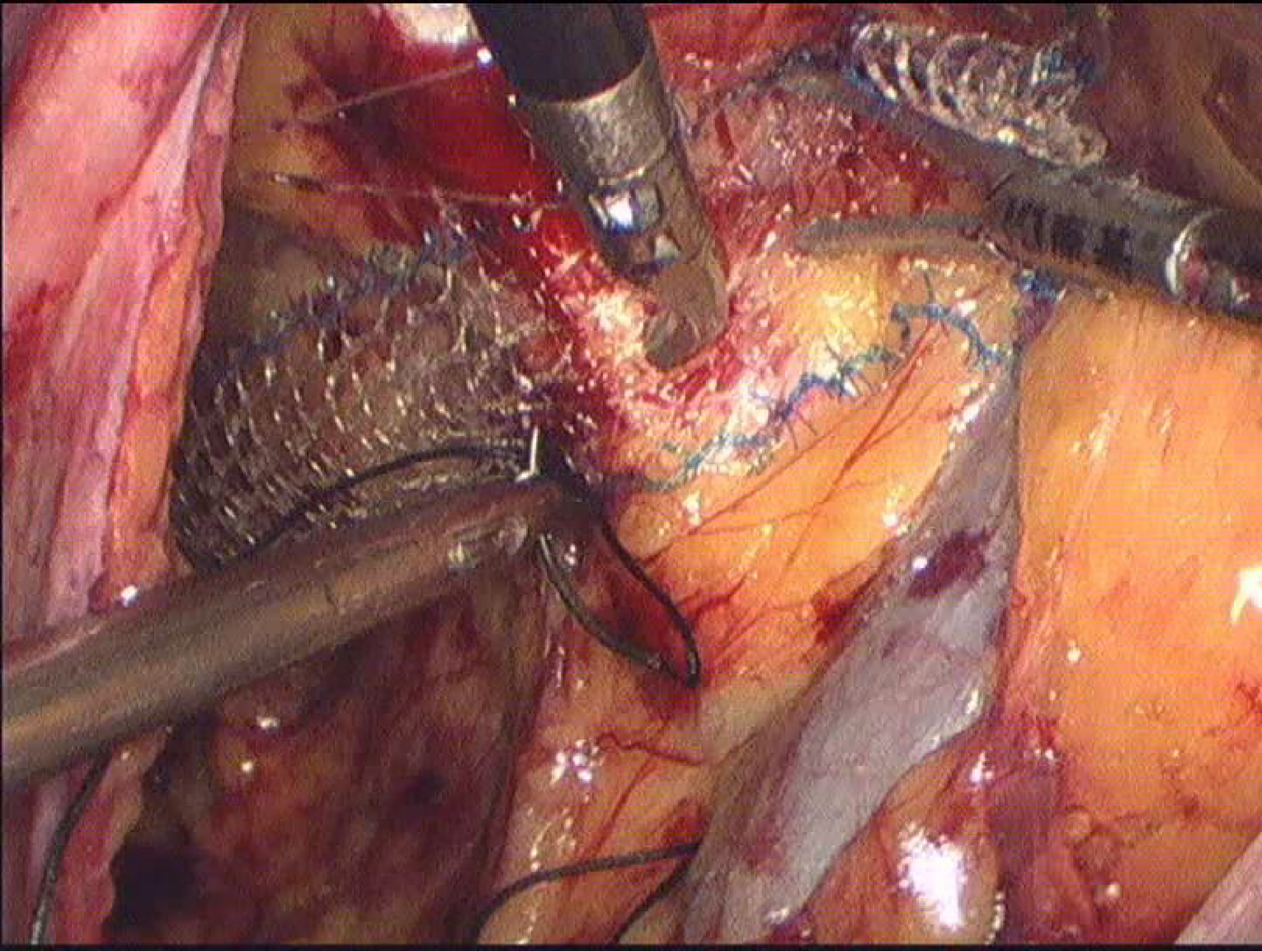
The mesh fixed with Cooper ligament.

Above the outside of the broken round ligament, we opened the side peritoneum and separated the connective tissue beneath it. The perivesical space was opened between the umbilical ligament and iliac vessels and their separation continued transversely and deeply. We exposed the Cooper ligament, obturator internus, and levator ani muscle and connected arcus tendineus fasciae pelvis (ATFP). Three types of expressions were observed during the operation: ATFP separation; the space between the obturator internus muscle and the levator ani muscle is loose and concave; and ATFP defect or separation from the levator ani muscle (Figure 2). We aligned strip-type polypropylene meshes (16 cm long and 1.5 cm wide) transversely above the gap, over the tendinous arch on the surface of levator ani muscle, covered the gap (Figure 3), and sutured the meshes and the tendinous arch on both sides of the gaps between the levator ani muscle and obturator internus. The tightness of the mesh was adjusted until both muscles could move properly when pulling the mesh. The other side of the mesh was sutured and fixed with the Cooper ligament (Figure 4) to keep the mesh tension-free. Excessive meshes were cut off, and the pelvic peritoneum was closed.

### 2.5. Observation

The patients were followed up at 3, 6, and 12 months after the operation.

The annual follow-up was completed in December 2020. Inquiry at follow-up pre- and post-operative symptoms (e.g., abdominal pain, falling pelvic cavity, urination, sexual activity) improved the situation by determining whether there is a feeling of vaginal mass prolapse; at the same time, the Quality of Life Questionnaire Score served as an evaluation index of subjective satisfaction; the package included the Pelvic Floor Dysfunction Questionnaire Short Form (PFDI-20), Pelvic Floor Function Impact Questionnaire Short form (PFIQ-7), and the Sexual Function Questionnaire Score (PIQS-31). The patient was satisfied to be free of pelvic pain and dysuria after the operation. Comparing post-operative scores with preoperative scores, the lower the PFDI-20 and PFIQ-7 scores, the more the satisfaction; the higher the PIQS-31 score, the higher the sexual satisfaction. POP-Q pelvic floor ultrasound examination of the genital tract hiatus area and bladder neck mobility was used to evaluate the objective cure rate. The objective cure rate is 0–I degree of the POP-Q score, and the recurrence rate is greater than II degree of the POP-Q score after the operation.

### 2.6. Statistical treatment

Data collection and statistical analyses were performed using IBM SPSS Statistics 24.0 software (IBM Corp., Armonk, NY, United States). All variables are presented as the mean and standard deviation or number (%). Categorical variables were compared by the chi-squared test. Pre-operative and post-operative scores of the POP-Q were compared using paired-sample *t*-tests. Results with a *P* value of <0.05 were considered statistically significant.

## 3. Results

The patients’ ages are in the range of 52–71, with an average of 63.28 ± 5.86; times of delivery are in the range of 2–6, with a BMI of 23.21 ± 0.73 kg/m^2^. Before the operation, POP-Q evaluation showed that 31 cases were at III and 45 cases were at IV. The six cases were found with SUI. GE Voluson E8 examination showed that the gap of the genital tract was 33.76 ± 0.73 cm^2^, and bladder neck displacement was 3.49 ± 0.28 cm. A summary of patient characteristics is shown in Table 1.

**Table 1:** General information.

Patient characteristics	
Variable	Values (*n* = 76)
Age (year ± SD)	63.28 ± 5.86
**Delivery [times, *N* (%)]**	
2	20 (26.30%)
3	25 (32.90%)
4	21 (27.60%)
5	9 (11.80%)
6	1 (1.30%)
BMI (kg/m^2^)	23.21 ± 0.73
SUI (*n*, %) POP-Q (pre-operation)	6 (7.8%)
III degree	31 (40.80%)
IV degree	45 (59.20%)
Follow-up (month)	20.64 ± 5.97

BMI, body mass index; POP, pelvic organ prolapse; SUI, stress urinary incontinence.

Values are mean ± standard deviations (range) or number (%).

All 76 patients went through the procedure successfully without any complications. The bleeding volume was 51.35 ± 20.94 ml, the ventilation time was 32.18 ± 4.38 h, and no medical complications were seen. Catheters were removed 3 days after operation, and the residue urine volume examination was normal except three patients who had difficulty urinating so their catheters were removed 6 days after the operation. Among the six patients with stress urinary incontinence (simultaneously operated on for TVT-O), no stress urinary incontinence was observed after the operation. The remaining 70 patients did not have stress urinary incontinence after the operation. A follow-up examination was carried out at 3, 6, and 12 months and annually afterwards. The longest follow-up visit lasted for 5 years, while the shortest lasted for 3 months, with an average period of 20.64 ± 5.97 months. The six cases had post-operational pelvic pain which lasted about 6 months. Mesh erosion was observed in one case (extrafascial hysterectomy). The excessive meshes were cut off through the vagina, and the patient recovered after using female hormone ointment. Post-operative POP-Q scores were between 0 and 1. Pelvic ultrasound examination 6 months after the operationshowed that the gap of the genital tract was 14.15 ± 0.55 cm^2^ and bladder neck displacement was 0.73 ± 0.15 cm. After 12 months, the gap of the genital tract was 14.16 ± 0.54 cm^2^ and bladder neck displacement was 0.74 ± 0.15 cm. Comparing pre- and post--operative pelvic ultrasound examination results, we observed statistically significant differences (*P* < 0.01). None of the 76 cases had relapsed (see Table 2). Post-operational subjective symptoms were improved compared with pre-operational conditions, such as abdominal pain and the feeling of pelvic cavity tenesmus, which was apparently weakened; there was no urethral dysfunction. Post-operational results of PFDI-20 and PFIQ-7 were evidently lower than pre-operational results, indicating a lower influence on living quality; post-operational results of PIQS-31 were higher than before operation, giving no statistical meaning but suggesting a higher level of satisfaction to sexual activities after (see Table 3).

**Table 2:** Comparing pre- and post-operation.

Time	POP-Q	The gap of genital tract (cm^2^)	Bladder neck displacement (cm)
Pre-operation	III: 31 (40.80%) IV: 45 (59.20%)	33.76 ± 0.73	3.49 ± 0.28
6 months after operation	0: 50 (65.80%) I: 26 (34.20%)	14.15 ± 0.55	0.73 ± 0.15
12 months after operation	0: 41 (53.90%) I: 35 (46.10%)	14.16 ± 0.54	0.74 ± 0.15
P1		0.012	0.004
P2		0.013	0.004
P3		0.000	0.000

POP, pelvic organ prolapse.

P1, pre-operation and 6 months after operation; P2, pre-operation and 12 months after operation; P3, 6 months after operation and 12 months after operation.

**Table 3:** Compare of the quality of life questionnaire.

Time	PFDI-20	PFIQ-7	PIQS-31
Pre-operation	78.9 ± 14.2	98.7 ± 18.5	49.1 ± 4.5
6 months after operation	12.4 ± 2.4	14.6 ± 2.8	53.2 ± 6.4
12 months after Operation	12.2 ± 2.2	14.4 ± 2.3	54.1 ± 6.7
*P*	0.002	0.001	0.387
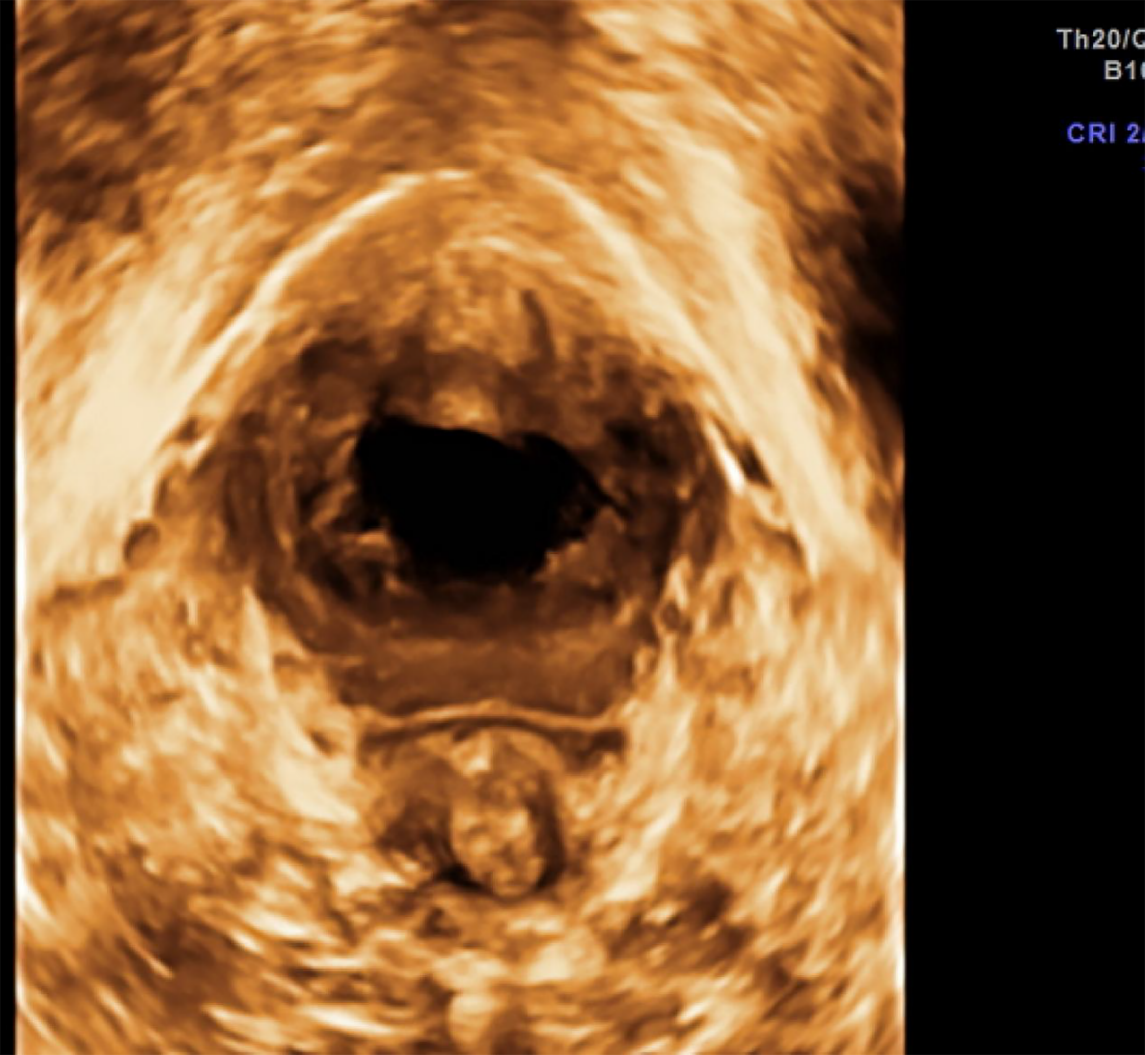 Genital tract (pre-operation)			
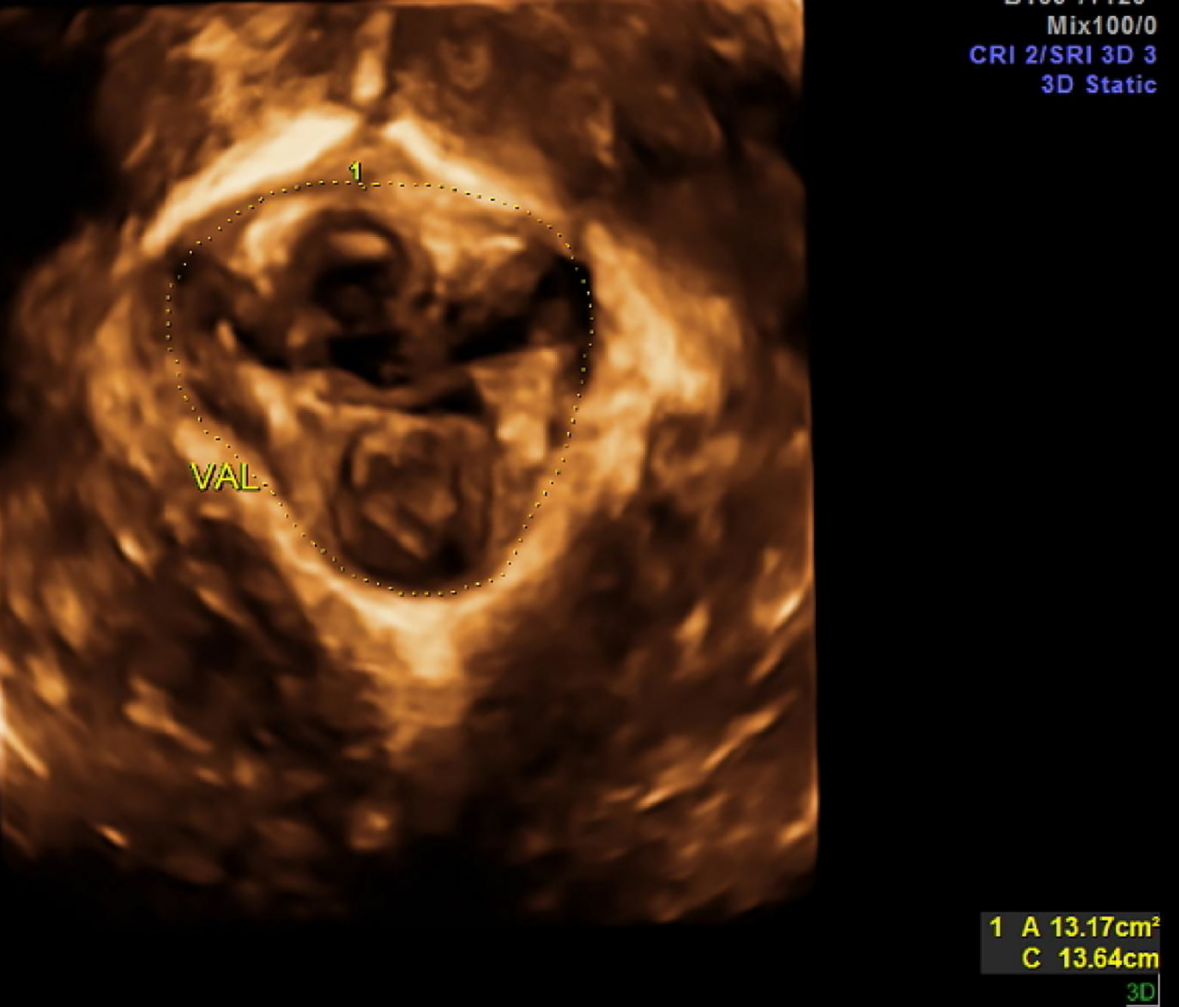 Genital tract (post-operation)			
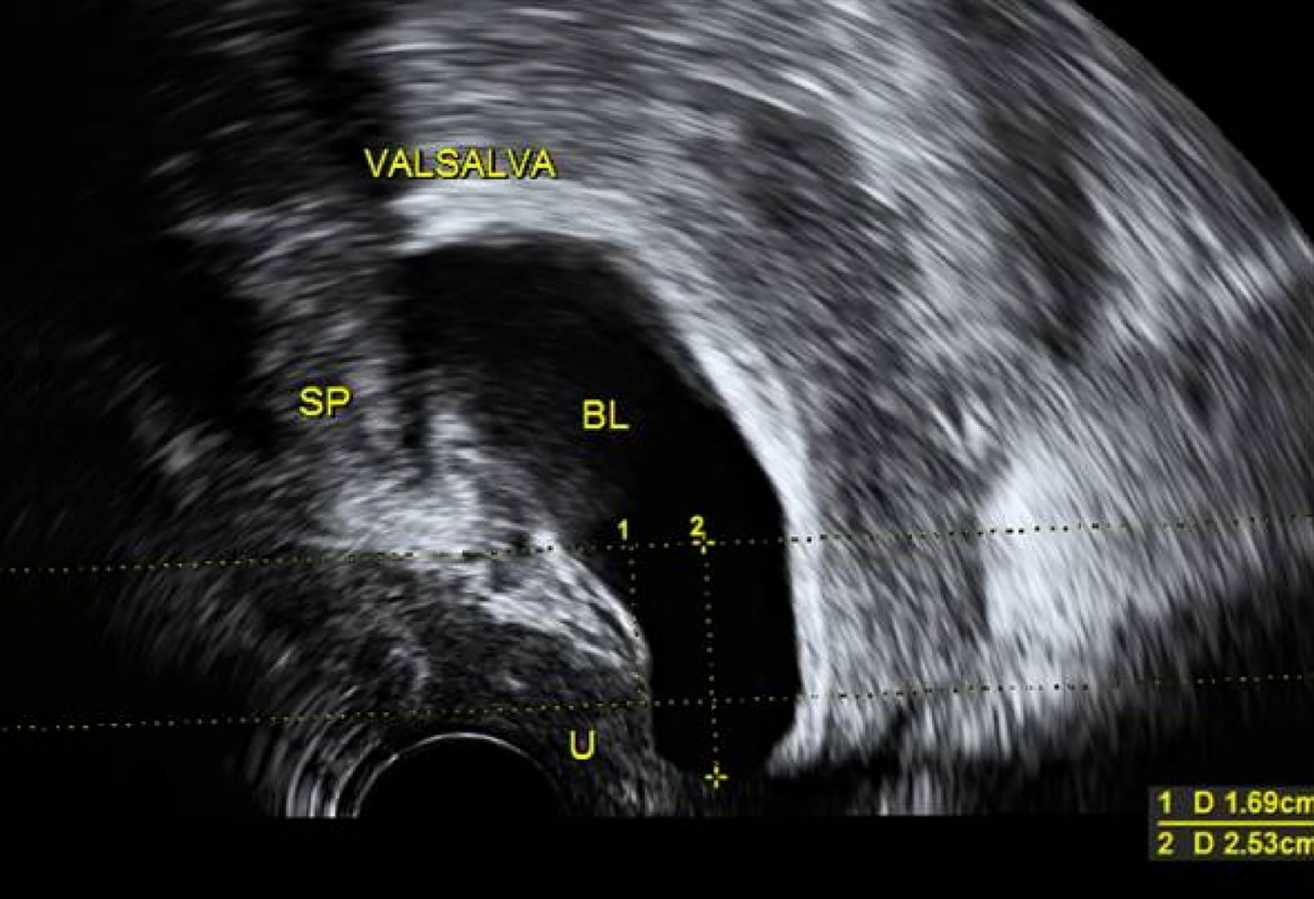 Pre-operation			
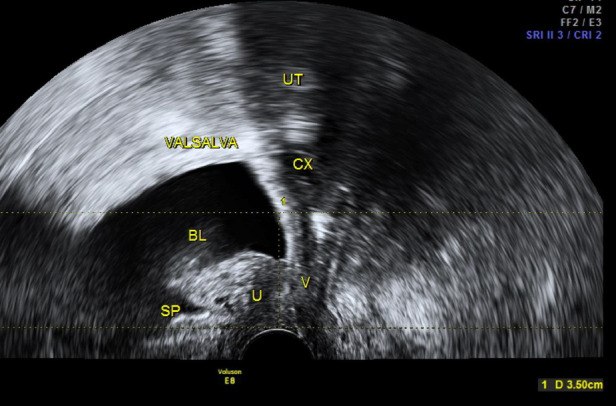 Post-operation			

## 4. Discussion

Current studies suggest that the main cause of POP is pelvic floor-associated tissue damage and the loss of integrity of the uterosacral ligament and the cardinal ligament complex. Therefore, the most thorough and effective means to treat such patients is surgical treatment. Vaginal hysterectomy and vaginal wall repair are two of the classic techniques; however, they have some disadvantages, such as easy recurrence after operation, where the recurrence rate can reach 20%–50% (12). In recent years, the development of materials science has made great advances in the surgical treatment of POP. A variety of biological synthetic grafts are used to strengthen the already weak tissue.

LSC is recognised as the “gold standard” operation for level I defects (13, 14); it reinforces the pelvic cavity, limits the mobility degrees of top of the vagina or cervix, can restore the vaginal axis, and maintain vaginal length. However, LSC merely corrects level I defects, the control of paravaginal force is weak, there is an increase in abdominal pressure, and the pelvic rear power strengthens. Because the control of the paravaginal force is not strengthened, abdominal pressure acting on the vagina breaks through the weak side, eventually leading to the relapse of anterior vaginal prolapse. Prolapse recurs in the anterior compartment in up to 10% of cases, depending on the follow-up time (15). Anterior recurrence after sacrocolpopexy is difficult to treat, especially in cases where the apex is still well suspended and the patient is sexually active. LSC is recognised as being equivalent to abdominal sacrocolpopexy (ASC) and robot-assisted laparoscopic colposacropexy (RALCS) and equivalent to or superior to transvaginal mesh (TVM) and other surgical procedures (16, 17); however, the costs of RALCS are significantly higher than those of LCS (18). Since the US Food and Drug Administration (FDA) stopped the distribution of transvaginal meshes, native tissue repair surgeries have been re-evaluated. Shull's technique of uterosacral ligament suspension (USLS) provides a safe and effective approach for apical prolapse without prosthetic materials (19). However, USLS resulted in a higher prolapse recurrence rate than sacrocolpopexy for stage III prolapse (20), and the risk of ureteral obstruction could not be totally avoided even if intraoperative cystoscopy was performed (21). There are no studies defining the best surgical technique for the treatment of second POP recurrence, and as a result, a standard procedure is still lacking. Vitale et al. have evaluated the effect of bilateral sacrospinous ligament fixation on post-operative recurrence (22), but this procedure can cause pelvic floor pain and affect patients' quality of life. Hence, we aimed to adapt our technique to avoid POP recurrence.

In this article, we strengthened level I through LSC, sacral ligament fusion, and Cooper ligament suspension and levels II and III through anterior and posterior vaginal repair. Cooper ligament suspension repairs the vaginal vault prolapse and anal levator fissure—the basin between wall defects, effectively controlling the excessive pelvic diaphragm displacement of the vagina. This new technique allows for a more effective suspension of the lateral part of the vagina and can lead to fewer anterior recurrences.

In our research, the post-operative POP-Q score was 0–I degree, the gap of the genital tract and bladder neck mobility decreased compared with those before the operation, and the differences were statistically significant (*P* < 0.01) 6 months and 12 months after the operation. There was no significant difference between 6 and 12 months after the operation (*P* > 0.05). None of the 76 cases had relapsed. The scores of PFDI-1 and PFIQ-7 at 6 and 12 months after the operation were significantly lower than those before the operation (*P* < 0.01), indicating that surgical treatment can improve the quality of life. There was no significant difference between PIQS-31 scores (*P* > 0.05). There was no significant improvement in sexual life; it was considered that the patients with POP are elderly, and the frequency of sexual life was significantly reduced.

We tried to choose intrafascial hysterectomy with preservation of the stereoscopic ring around the cervix (23), and according to the degree of sacral ligament damage, we decided on the sacral ligament fusion area. While maintaining the advantage of sacral fixation by sacral ligament fusion to repair and strengthen the sacral ligament relaxation, the pelvic fixation should be restored. Because the mesh and sacral ligament had no tension on the right side of the implants, we strengthened the intensity of the sacral ligament and fusion of sacral ligament back to proper tension, closed the retroperitoneum and pelvic peritoneum, and greatly reduced the chances of mesh erosion. We avoided direct suturing of the mesh to the end of the vagina to reduce the mesh exposure to erosion and the occurrence of chronic pelvic pain in patients.

The extrafascial uterus, the cardinal ligament, and the sacral ligament were removed from the centre of the pelvic floor adjacent tissues so that the pelvic fascia, adjacent tissues, and other structures underwent the same degree of alteration, which weakens the pelvic floor supporting structure and is likely to increase the risk of post-operative recurrence. Intrafascial hysterectomy should preserve the cardinal ligament and sacral ligament structures, requiring the surgeon to separate the anatomical structures more carefully to avoid the destruction of the pericervical stereoscopic ring, but it did not increase operation time or affect post-operative recovery. After 6 months, we have only one case of mesh erosion (fascia hysterectomy) (1.32%) in our patients, which was cured after vaginal excision and oestrogen ointment. After improvement, the patch suture anchor point is selected at the beginning of the sacral ligament, focusing on the ligament, rather than the end of the vaginal cuff; again, there is no mesh erosion.

Although sacrocolpopexy is described as one of the safest procedures for the surgical treatment of prolapse (24), surgeons should be aware of the potential risk of spondylodiscitis caused by sacrocolpopexy. We should carefully place the presacral fixation and put stitches or tacks into the anterior longitudinal ligament, avoiding the disc space, to minimise the risk of spondylodiscitis (25).

A balanced control of the pelvic floor centred on preserving the stereoscopic ring around the cervix has many advantages. First, to ensure the completion of the pelvic floor structure by retaining the three-dimensional ring around the cervix integral, the combination of whole pelvic floor repair, pelvic anatomy restoration, and normal function restoration was achieved. Second, at the same time, it is suspended laterally to balance the pelvic floor force, make three horizontal reinforcements, limit the pelvic viscera in a mobile range, andavoid a weak side that would then result in recurrence of prolapse. Third, using a self-cut L-mesh, only on the cervical ring structure, will not place the mesh into the vagina, causing erosion or stiffness of the mesh, thereby reducing the patient's occurrence of pelvic pain and improving the post-operative quality of life. So, we consider that pelvic floor functional restoration is to repair the damage and reinforcing strength rather than a simple suspension. The application of a mesh is to repair the damage and reinforce the tendon and ligament bow itself, making the organs mobile; therefore, the pelvic floor reconstruction surgery restored the normal anatomical position and function.

We reviewed the reports after 2 years of LSC; the objective success rate was 92%, the subjective success rate was 94.4%, the prolapse of the reoperation rate was 6.2%, the mesh exposure rate was 2.7%, the post-operative sexual dysfunction rate was 7.8%, the voiding dysfunction rate was 18.2%, and the intestinal dysfunction rate was 9.8% (26). Through clinical observation and follow-up of 5 years, it was found that there was no recurrence, including in six patients with stress urinary incontinence after surgery. There was no dysuria or intestinal dysfunction. Patients' quality of life questionnaire scores showed 100% post-operative satisfaction.

This study had several strengths. First, to the best of our knowledge, this is a study with a balanced control of the pelvic floor centred on the preservation of the stereoscopic ring around the cervix during revascularization. We hope that these data will help provide more counselling for the development of this new surgical approach. Second, the surgeries were completed by the same medical team. The post-operative vaginal examination was performed by the chief surgeon. These methods prevented the influence of variations across different surgeons.

However, this study has some limitations that should be considered. First, this was a single-centre retrospective study with a limited sample size. Second, the lack of a control group (including patients undergoing different types of surgical procedures) is another limitation of our study. More patients need to be assessed.

## 5. Conclusion

A balanced control of the pelvic floor centred on the preservation of the stereoscopic ring around the cervix through revascularization may be a valuable choice for treating POP with low risks of prolapse recurrence as well as good safety and efficacy. However, this concept should be confirmed with larger randomised controlled trials and longer follow-up times.

## Data Availability

The raw data supporting the conclusions of this article will be made available by the authors without undue reservation.
